# Innate Immune Signalling Genetics of Pain, Cognitive Dysfunction and Sickness Symptoms in Cancer Pain Patients Treated with Transdermal Fentanyl

**DOI:** 10.1371/journal.pone.0137179

**Published:** 2015-09-02

**Authors:** Daniel T. Barratt, Pål Klepstad, Ola Dale, Stein Kaasa, Andrew A. Somogyi

**Affiliations:** 1 Discipline of Pharmacology, School of Medicine, University of Adelaide, Adelaide, Australia; 2 Department of Circulation and Medical Imaging, Norwegian University of Science and Technology, Trondheim, Norway; 3 Department of Anaesthesiology and Intensive Care Medicine, St Olavs University Hospital, Trondheim, Norway; 4 Department of Cancer Research and Molecular Medicine, European Palliative Care Research Centre, Norwegian University of Science and Technology, Trondheim, Norway; 5 Department of Clinical Pharmacology, Royal Adelaide Hospital, Adelaide, Australia; 6 Centre for Personalised Cancer Medicine, University of Adelaide, Adelaide, Australia; Central South University, CHINA

## Abstract

Common adverse symptoms of cancer and chemotherapy are a major health burden; chief among these is pain, with opioids including transdermal fentanyl the mainstay of treatment. Innate immune activation has been implicated generally in pain, opioid analgesia, cognitive dysfunction, and sickness type symptoms reported by cancer patients. We aimed to determine if genetic polymorphisms in neuroimmune activation pathways alter the serum fentanyl concentration-response relationships for pain control, cognitive dysfunction, and other adverse symptoms, in cancer pain patients. Cancer pain patients (468) receiving transdermal fentanyl were genotyped for 31 single nucleotide polymorphisms in 19 genes: *CASP1*, *BDNF*, *CRP*, *LY96*, *IL6*, *IL1B*, *TGFB1*, *TNF*, *IL10*, *IL2*, *TLR2*, *TLR4*, *MYD88*, *IL6R*, *OPRM1*, *ARRB2*, *COMT*, *STAT6* and *ABCB1*. Lasso and backward stepwise generalised linear regression were used to identify non-genetic and genetic predictors, respectively, of pain control (average Brief Pain Inventory < 4), cognitive dysfunction (Mini-Mental State Examination ≤ 23), sickness response and opioid adverse event complaint. Serum fentanyl concentrations did not predict between-patient variability in these outcomes, nor did genetic factors predict pain control, sickness response or opioid adverse event complaint. Carriers of the *MYD88* rs6853 variant were half as likely to have cognitive dysfunction (11/111) than wild-type patients (69/325), with a relative risk of 0.45 (95% CI: 0.27 to 0.76) when accounting for major non-genetic predictors (age, Karnofsky functional score). This supports the involvement of innate immune signalling in cognitive dysfunction, and identifies MyD88 signalling pathways as a potential focus for predicting and reducing the burden of cognitive dysfunction in cancer pain patients.

## Introduction

Common symptoms of cancer and its treatment are themselves a major health burden; chief among these is pain [1]. Opioids are the mainstay of cancer pain management, but many cancer patients experience inadequate pain relief and/or adverse opioid effects that reduce their quality of life [2,3].

Transdermal fentanyl is a strong opioid analgesic targeted to patients with stable opioid requirements [4]. It is commonly prescribed for the treatment of moderate to severe cancer pain [3], but has similar limitations to oral morphine with respect to inadequate pain control and adverse effects [5]. Clinical experience shows that individualisation is the key to successful opioid treatment. This is due to opioids’ narrow therapeutic index, as well as the large interindividual variability in their pharmacokinetics (PK) and pharmacodynamics (PD), and patients’ pain sensitivity, phenotype, perception and acceptance. Genetics can contribute to variability in these factors and thus variable opioid response [6].

The European Pharmacogenetic Opioid Study (EPOS) was a multinational collaborative effort to identify factors, particularly genetic, that determine opioid requirements for moderate-severe cancer pain [7]. In a sub-analysis of EPOS data, Klepstad et al. (2011) showed that transdermal fentanyl dose requirements for cancer pain are not associated with genetic variability in classical opioid and neuronal signalling genes, but with polymorphisms in *STAT6*; a cytokine- and growth factor-responsive transcription activator [7]. Those findings raise the question of whether genetic variability in non-classical opioid signalling pathways, in particular those involved in immune reactivity, might impact on fentanyl response in cancer pain patients.

There is growing evidence that opioids including fentanyl, as well as endogenous damage-associated molecular patterns (DAMPs) and peripheral inflammation associated with tissue damage from cancer or chemotherapy, can activate central innate immune cells (i.e. glia) via the toll-like receptor (TLR) family of pattern recognition receptors (e.g. TLR4) [8–11]. Activated glia release cytokines, chemokines, DAMPS and other inflammatory mediators that activate other glia, can facilitate peripheral immune cell infiltration into the CNS, and modify neuronal activity [12]. For example, immune mediators released by activated glia and infiltrating peripheral immune cells can enhance neuronal excitability, leading to central sensitisation and increased nociceptive signalling. This commonly manifests as hyperalgesia and/or allodynia that adds a pathological component to physiological pain associated with tissue (especially neuronal) damage, and is a major barrier to effective long-term opioid analgesia [9,10,12].

Other common symptoms experienced by cancer pain patients (e.g. nausea, fatigue, depression) are highly correlated and characteristic of cytokine-induced sickness responses, suggesting a shared neuroinflammatory mechanism [13]. Proinflammatory cytokine administration has also been shown to induce cognitive dysfunction in mice, and inflammatory cytokine immunotherapy is associated with significantly greater incidence of cognitive dysfunction in cancer patients when compared to primarily cytotoxic agents [14].

Therefore, we hypothesise that genetic variability in innate immune activation and inflammation pathways contributes to between-patient variability in pain control and adverse symptoms among cancer pain patients receiving transdermal fentanyl. The aim of this study was to investigate if common polymorphisms in genes involved in innate immune activation, inflammatory signalling, and consequent neuronal adaptation, alter the serum fentanyl concentration-response relationships for pain control, cognitive dysfunction, and adverse symptom complaint (sickness response and opioid side-effects), in EPOS cancer pain patients on transdermal fentanyl.

## Materials and Methods

### Subjects

EPOS is a multicentre (17 centres in 11 European countries) collaborative study of 2294 cancer patients with a malignant disease treated with an opioid for moderate to severe pain (step III at the WHO treatment ladder for cancer pain [15]). All EPOS patients were over 18 years of age, had a verified malignant disease, and were treated with regular opioids for their cancer pain for a minimum duration of 3 days. EPOS was conducted in accordance with the Declaration of Helsinki, the protocol was approved at each study centre’s local ethics committee (Regional Medical Research Ethics Committee, Central Norway Health Authority, Protocol reference number: 119–03, approved 27.09.03), and each participant provided written, informed consent [7].

Subjects selected for this study analysis were a subset of participants from EPOS maintained on transdermal fentanyl for pain control.

### Sample selection

Of EPOS participants, 728 were treated with transdermal fentanyl and shortlisted for the current study. Of these, patients were excluded from analysis because they received non-transdermal fentanyl for break-through pain during the study period (n = 15), were missing blood samples (n = 6), were missing data on serum fentanyl concentrations (n = 9), were missing samples for genetic evaluation (n = 8) and/or had an unclear fentanyl dose (n = 14). The PK pharmacogenetics of the remaining 676 patients has previously been published by the authors [16]. For the present study, patients were additionally excluded if they were scheduled other opioids in addition to fentanyl (n = 110) and/or were non-Caucasian (n = 10). Patients who received breakthrough opioids other than fentanyl, or weak opioids, in the previous 24 hours were not excluded. Of the remaining 556 patients, 468 had data required for analysis of at least one outcome measure (394 for all outcome measures) and were included in the final study analyses.

### Data collection from EPOS

Relevant single time-point patient data used for the present study are outlined in Table 1, in addition to average pain assessed using the Brief Pain Inventory (BPI) [17]; and cognitive function assessed using the Mini-Mental State Examination (MMSE) [18,19]. Genetic clustering of EPOS patients into 4 main European ancestry subgroups was previously determined [20]. A complete list of patient data taken as part of the original EPOS study is in [7].

**Table 1:** Patient characteristics and investigated non-genetic variables (for n = 468 included in analyses).

Variable	n	Median ± SD (range) or counts.	Analysis notes^b^
Age	468	64 ± 12 (24–88)	λ = 2 (squared) transformation
Sex	468	218 Male / 250 Female	
Treatment centre country	468	Switzerland = 19; Germany = 109; Denmark = 1; Finland = 7; United Kingdom = 13; Greece = 3; Iceland = 45; Italy = 148; Lithuania = 38; Norway = 71; Sweden = 14.	
BMI (kg/m^2^)	460	23 ± 5 (9–41)	Log_10_ transformed
Serum albumin concentration (g/L)	445	33 ± 7 (11–67)	λ = 0.75 transformation
Serum C-reactive protein concentration (mg/L)	467	≤ 40 mg/L = 264; > 40 mg/L = 203	
Creatinine clearance (Cockcroft–Gault[21]: mL/min)	459	84 ± 45 (13–308)	λ = 0.25 transformation
Kidney Disease	468	29	
Time on opioids (days)	438	52 ± 272 (2–2332)	Log_10_ transformed
Fentanyl patch delivery rate^a^ (μg/h)	468	50 ± 53 (12.5–400)	
Serum fentanyl concentration[22] (nM)	468	5.6 ± 11.1 (0.09–144.5)	Log_10_ transformed
Cancer diagnosis	468	Haematological = 18; Breast = 51; Prostate = 34; Urological = 36; Lung = 70; Gastrointestinal = 104; Female reproductive = 57; Sarcoma = 15; Head and neck = 37; Pancreatic = 19; Skin = 7; Liver = 5; Mesothelioma = 5; Unknown origin = 13.	
Metastases	468	Liver = 125; Bone = 176; CNS = 26; Lung = 111.	
Co-medications in previous 24 hours	468	Breakthrough opioid = 159 (oral morphine = 133; subcutaneous morphine = 58; oral oxycodone = 41; intravenous morphine = 23; subcutaneous ketobemidone = 6; oral hydromorphone = 1); Gabapentin = 83; Weak opioid = 41; Systemic glucocorticoid = 225; Paracetamol = 79; Benzodiazepine = 127; NSAID = 140; Hypnotic = 70.	
Total breakthrough opioid dose in previous 24 hours^a^ (mg, oral morphine equivalent)	159	30 ± 77 (5–580)	
Pain category	468	Visceral = 91; Bone and soft tissue (deep somatic) = 168; Neuropathic = 24; Mixed = 184; Unknown = 1.	
Pain location	468	Head = 62; Thoracic/upper abdominal = 170; Pelvic = 188; Back = 252; Upper extremity = 65; Lower extremity = 116.	
Karnofsky Performance Status Scale[23]	467	60 ± 17 (20–100)	Square root transformed
EORTC QLQ-C30: Nausea and vomiting symptom scale	425	<50 = 336; ≥ 50 = 89	
EORTC QLQ-C30: Constipation symptom scale	422	<50 = 244; ≥ 50 = 178	
EORTC QLQ-C30: “Were you tired?”	424	“Not at all” or “A little” = 175; “Quite a bit” or “Very much” = 249	
EORTC QLQ-C30: “Did you feel depressed?”	421	“Not at all” or “A little” = 286; “Quite a bit” or “Very much” = 135	

^a^Not included as a non-genetic variable.

^b^Data transformations to a normal distribution used to for regression analysis [λ represents Box-Cox transformation: (x^λ^-1)/λ].

BMI: body mass index. EORTC QLQ-C30: European Organisation for Research and Treatment of Cancer Quality-of-Life Questionnaire-C30 [24].

### Genotyping

DNA was extracted from EDTA–treated whole blood [7]. Patients were genotyped for the following 20 SNPs in 14 genes using a custom Sequenom MassARRAY (iPLEX GOLD) multiplex panel designed and implemented at the Australian Genome Research Facility (Brisbane, Australia) [25]: Innate immune activation–*TLR4* (rs4986790, rs4986791); *TLR2* (rs3804100); *MD2* (*LY96*) (rs11466004); *MYD88* (rs6853). Inflammatory mediators–*IL1B* (rs1143627, rs1143634, rs16944); *CASP1* (*ICE*) (rs554344, rs580253); *IL6* (rs10499563); *IL6R* (rs8192284); *IL10* (rs1800871, rs1800896); *IL2* (rs2069762); *CRP* (rs2794521); *TGFB1* (rs11466314, rs1800469); *TNFA* (rs1800629). Neuronal adaptation—*BDNF* (rs6265).

Reproducibility of the multiplex panel was confirmed by re-genotyping 100 randomly selected patients (99% for rs1143634 and 100% for all other SNPs).

*COMT* (rs4680), *OPRM1* (rs1799971), *ARRB2* (rs3786047, rs1045280, rs2271167, rs2036657), *STAT6* (rs3024971, rs167769) and *ABCB1* (rs1045642, rs2235013, rs1128503, rs4437575, rs2235033, rs1202170, rs7802773) SNPs had been genotyped previously [7]. For *ABCB1*, only rs1045642, rs2235013 and rs1128503 were included in the final analysis based on existing evidence of phenotype associations and near complete linkage disequilibrium (LD) (r^2^ ≥ 0.9) with the other *ABCB1* SNPs.

Details of SNP locations and nucleotide/amino acid changes are provided in S1 Table.

### Data analysis

Data were analysed using the R statistical program [26] unless indicated otherwise. Chi-squared analysis was used to test for genotype deviations from Hardy-Weinberg Equilibrium.

Patient descriptive data were summarised as median ± standard deviation (SD) and range (minimum to maximum), or as counts, as appropriate.

#### Measures of fentanyl response

Pathological, physiological and genetic variables were investigated for their association with four measures of fentanyl response: “pain control”; “cognitive dysfunction”; “sickness response”; and “opioid adverse event complaint”.

Patients with average pain of 3 or less measured on an 11-point NRS in the BPI were categorized as having “pain control”; higher scores were categorized as unacceptable pain [27]. Patients with a total MMSE of 23 or less were categorised as “cognitive dysfunction” [18,27]. Patients who reported two or more of the following were categorised as “sickness response”: nausea ≥ 50 (EORTC QLQ-C30 nausea and vomiting scale); tiredness ≥ 3 (EORTC QLQ-C30 item “Were you tired?”); and depression ≥ 3 (EORTC QLQ-C30 item “Did you feel depressed?”).

Based on previous EPOS studies, patients were categorised as “opioid adverse event complaint” if they reported nausea ≥ 50 (EORTC QLQ-C30 nausea and vomiting scale); constipation ≥ 50 (EORTC QLQ-C30 constipation scale); tiredness ≥ 3 (EORTC QLQ-C30 item “Were you tired?”); and/or had a total MMSE of 23 or less (“cognitive dysfunction”) [19,27,28].

The co-incidence of specific adverse events (nausea, tiredness, constipation, depression and cognitive dysfunction) was investigated using Fishers Exact Test.

Distributions of continuous variables were assessed using histograms and quantile-quantile (Q-Q) plots. If continuous variables were not normally distributed, optimal transformations to normalise the distributions were identified using the boxcox function in the MASS package [29]. Transformed data were then used in all subsequent analyses.

#### Identification of response predictors

The absence of significant associations between responses and ancestral subgroup was first confirmed by chi-squared analysis (P > 0.05) before proceeding with further analyses.

Details of the subsequent statistical analysis pipeline are provided in S1 Protocol. Briefly, major non-genetic variables (listed in Table 1) to be controlled for in subsequent genotype analyses were identified by Lasso regression, including first order interactions with serum fentanyl concentration. A step-down regression model selection procedure based on cross-validation error was used to identify genetic factors associated with different responses, fixing major non-genetic predictors as the base model. Epistasis was also investigated by generalised multifactor dimensionality reduction (GMDR) analysis, incorporating major non-genetic predictors into the response score.

Given the number of genetic factors investigated, the likelihood of associations occurring by chance within the data for each outcome measure were investigated by comparing the performance of the final regression and GMDR models against control models using randomised permutations of paired response and non-genetic variable data.

To evaluate the data analysis approach of the current study against previously published EPOS findings [7], the analysis approach was also applied to identify predictors of fentanyl delivery rate (previously associated with *STAT6* rs167769).

## Results

### Genetic variability

Allele and genotype frequencies for each SNP are given in S1 Table. No genotype frequencies significantly deviated from Hardy Weinberg Equilibrium (P > 0.1). Details of SNP linkage disequilibrium and haplotypes are given in S1 Text.

There was no significant association between ancestral subgroups and any response (point-wise P > 0.05).

### Pain control

Of 430 patients with BPI scores, 210 were classified as having pain control. No major non-genetic predictors of pain control were identified by Lasso regression, including serum fentanyl concentrations (median ± SD in pain relieved = 5.4 ± 7.7 μM versus not pain relieved = 5.8 ± 13.6 μM). *CRP* rs2794521 variant genotype was associated with reduced pain control (optimal k = 2.4, CVE = 0.250 < base model = 0.251), however cross-validation performance of this model was no better than randomised controls (median (25–75^th^ percentile) CVE = 0.249 (0.247–0.251)). Reflecting this, the predictive performance of the model was very poor (area under the ROC curve = 0.53), and basic chi-squared analysis not significant (χ^2^ = 3.6, point-wise P = 0.17).

No epistatic models for pain control performed better than randomised dataset controls.

*STAT6* rs167769 was the sole predictor of fentanyl delivery rate, but fentanyl delivery rate was not a predictor of between-patient variability in pain control (median ± SD delivery rate in pain controlled = 50 ± 47 μg/hr versus unacceptable pain = 50 ± 54 μg/hr).

### Cognitive dysfunction

Of 438 patients with MMS data, 81 had cognitive dysfunction. Analysis of the co-incidence of specific adverse events showed cognitive dysfunction was unrelated to other adverse events, but there were significant positive associations between nausea, tiredness, depression and constipation (Table 2).

**Table 2:** Co-incidence of adverse events reported by cancer pain patients receiving transdermal fentanyl.

OR (95% CI)	Nausea	Tiredness	Depression	Constipation
**Tiredness**	5.5 (3.0 to 10.4)****	-	-	-
**Depression**	1.7 (1.1 to 2.8)*	4.0 (2.5 to 6.4)****	-	-
**Constipation**	2.0 (1.3 to 3.2)**	2.0 (1.3 to 3.0)***	1.8 (1.2 to 2.7)**	-
**Cognitive Dysfunction**	0.83 (0.44 to 1.6)	0.81 (0.50 to 1.3)	1.3 (0.78 to 2.2)	1.1 (0.68 to 1.9)

*P<0.05

**P < 0.01

***P < 0.001

****P<0.0001 Fisher’s exact test

OR: Odds Ratio. CI: Confidence Interval.

**Fig 1:**
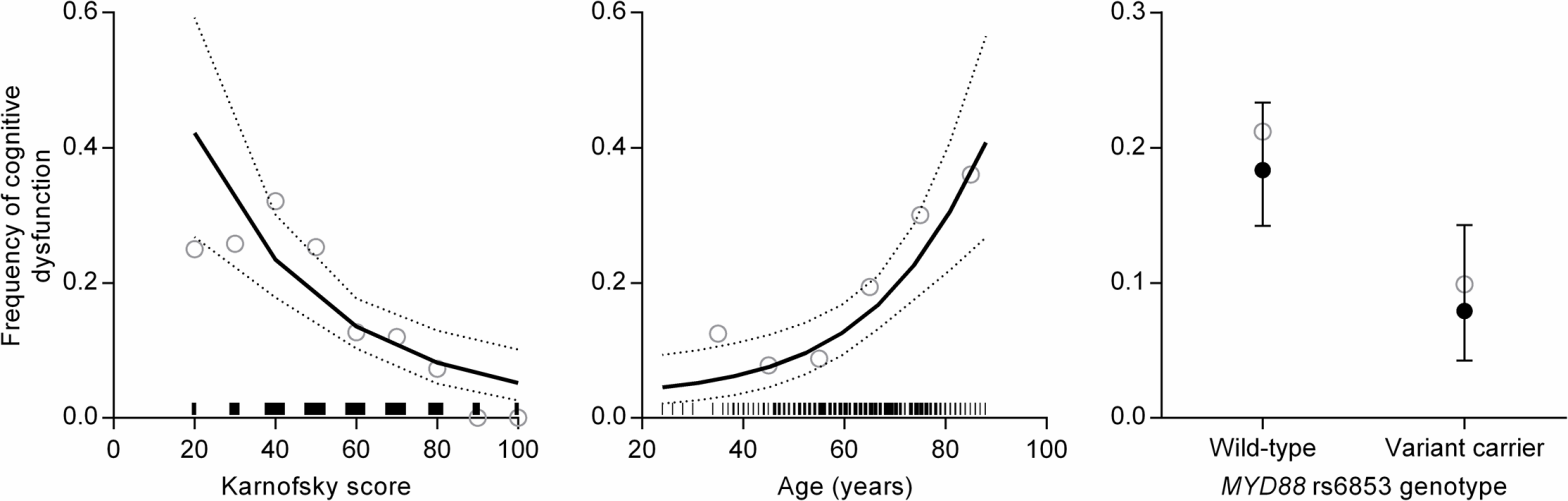
Predictors of cognitive dysfunction in cancer patients receiving transdermal fentanyl. Solid lines and filled circles are predicted frequency, and dotted lines and error bars are 95% confidence intervals, holding other variables to typical values. Hollow circles are unadjusted (raw) frequencies for each Karnofsky score, within 10-year bins from 30 years of age, or for each *MYD88* rs6853 genotype group. Vertical bars above the x-axes of Karnofsky score and Age represent the distributions of patients’ Karnofsky scores and ages, respectively.

Serum fentanyl concentrations were not associated with cognitive dysfunction (median ± SD in cognitive dysfunction = 7.1 ± 7.9 μM versus not cognitive dysfunction = 5.5 ± 11.3 μM). Older age and lower Karnofsky functional status were associated with increased cognitive dysfunction, with a modest predictive value (area under the ROC curve = 0.71). *MYD88* rs6853 heterozygous and variant genotypes (combined) were associated with reduced cognitive dysfunction (Table 3 and Fig 1) (optimal k = 7.4). Addition of *MYD88* rs6853 genotype (variant carrier versus non-carrier) slightly increased the predictive ability over the non-genetic model (area under the ROC curve = 0.73), and cross-validation performance of this model (CVE = 0.136) was better than randomised controls [median (25–75th percentile) CVE = 0.138 (0.138–0.138)]. The incidence of cognitive dysfunction in *MYD88* rs6853 variant carriers was less than half that of wild-type patients [11/111 (10%) versus 69/325 (21%), respectively), with a relative risk of 0.45 when accounting for age and Karnofsky functional score (Table 3 and Fig 1).

**Table 3:** Variables associated with cognitive dysfunction in cancer pain patients receiving transdermal fentanyl.

Regressor	Adjusted Odds Ratio^a^ (95% CI)		Nested model P-value	Relative risk^a^ (95% CI)
**(Intercept)**	1.65	(0.23 to 11.67)		
**Age** ^**2**^	1.00037	(1.00020 to 1.00056)	3.0 x 10^−5^	1.00029 (1.00018 to 1.00041)
**√Karnofsky score**	0.63	(0.49 to 0.79)	6.4 x 10^−5^	0.71 (0.62 to 0.82)
***MYD88* rs6853 variant carrier** ^**b**^	0.38	(0.18 to 0.73)	0.003	0.45 (0.27 to 0.76)

^a^Odds Ratio controlling for all other regressors.

^b^Homozygous wildtype genotype as reference.

Odds ratio and relative risk greater than 1 indicates an association with increased likelihood of cognitive dysfunction. √ = square root.

No epistatic models for cognitive dysfunction performed better than randomised dataset controls.

### Sickness response

Of 418 patients with EORTC data, 148 were classified as “sickness response”. Serum fentanyl concentrations were not associated with sickness response (median ± SD in sickness response = 5.4 ± 8.0 μM versus not sickness response = 5.6 ± 12.6 μM). The use of breakthrough opioids was associated with increased, and male sex and an Italian treatment centre were associated with decreased, sickness response with modest predictive value (area under the ROC curve = 0.69).

The *BDNF* rs6265 variant was associated with increased sickness response (S2 Table) (optimal k = 3.9). However, adding this genetic factor only slightly improved predictive ability over the non-genetic model (area under the ROC curve = 0.70), and cross-validation performance of this model (CVE = 0.208) was not better than randomised controls [median (25–75th percentile) CVE = 0.210 (0.207–0.210)].

The incidence of sickness response was not significantly different between *MYD88* rs6853 homozygous wild-type and variant carrier patients (67% versus 69%, respectively. OR (95% CI) = 1.1 (0.69 to 1.8)).

No epistatic models for sickness response performed better than randomised dataset controls.

### Opioid adverse event complaint

Of 430 patients with adverse event data, 329 were classified as opioid adverse event complainers. Serum fentanyl concentrations were not associated with opioid adverse event complaint (median ± SD in complainers = 5.8 ± 9.2 μM versus non-complainers = 5.1 ± 16.1 μM). Depression and the use of breakthrough opioids were associated with increased complaint with modest predictive value (area under the ROC curve = 0.69). *CASP1* rs554344 homozygous variant genotype and *ARRB2* variant diplotype were associated with increased, and *TGFB1* rs1800469 homozygous wildtype genotype associated with reduced, complaint (S3 Table) (optimal k = 2.1). Addition of these genetic factors increased the predictive ability over the non-genetic model (area under the ROC curve = 0.73), however cross-validation performance of this model (CVE = 0.157) was no better than randomised controls [median (25–75th percentile) CVE = 0.157 (0.156–0.159)].

No epistatic models for opioid adverse event complaint performed better than randomised dataset controls.

## Discussion

This study set out to investigate if polymorphisms in genes implicated in neuroimmune activation alter the serum fentanyl concentration-response relationship in cancer pain patients. However, serum fentanyl concentrations did not predict between-patient variability in pain intensity or adverse events, reflecting similar previous findings with oral morphine [27]. This further emphasises the major hurdles to effective opioid use for cancer pain: large underlying heterogeneity in cancer pain phenotypes, a milieu of cancer/chemotherapy adverse effects, and unpredictable between-patient variability in opioid pharmacodynamics. Given the absence of clear serum fentanyl concentration-response relationships, the direct relationships between variables (genetic and non-genetic) and pain and adverse symptoms were investigated.

No major predictors of pain intensity were detected in this study, despite investigating variables encompassing both underlying pain and fentanyl pharmacodynamics. The data analysis approach of the current study was able to identify *STAT6* rs167769 as a predictor of fentanyl dose as in a previous EPOS study [7], but dose itself (like serum concentrations) was not a predictor of between-patient variability in pain intensity.

Alternatively, a major genetic predictor of cognitive dysfunction in cancer pain patients receiving transdermal fentanyl was identified. Patients carrying the *MYD88* rs6853 variant allele had half the risk of developing cognitive dysfunction: an effect seen with and without accounting for non-genetic variables.

MyD88 is an adapter protein for multiple TLRs (e.g. TLR4 and TLR2) and the interleukin 1 receptor (IL1R) [30,31]. Therefore, it plays a central role in both the initial innate immune activation by opioids and/or DAMPs, and the potentiation of immune activation via IL1B signalling. TLR4/MyD88 signalling is important for the development of post-operative- and post-traumatic brain injury-induced cognitive dysfunction in rats and mice [32–34], and glial modulators are currently being trialled for the potential to attenuate post-operative cognitive dysfunction [35]. Thus it is likely that MyD88 signalling is also key to cognitive dysfunction in cancer pain patients, but to our knowledge this has not been specifically investigated to date. Whilst the direct functional effects of the rs6853 SNP, located within the 3’ untranslated region of *MYD88*, are not currently known, it has been associated with decreased vaccine response and increased susceptibility to infection [36,37], as well as electroencephalogram spindle amplitude during anaesthesia [25]. Therefore, we hypothesise that cancer pain patients carrying the rs6853 SNP are at lower risk of developing cognitive dysfunction due to reduced MyD88 activity driving the neuroimmune signalling associated with cognitive impairment. Polymorphisms of more specific signalling systems (e.g. *TLR4* and *IL1B*) were not significantly associated with cognitive dysfunction, indicating that the mechanistic importance of MyD88 across multiple cell types in parallel and sequential signalling systems may make it of specific importance to impairment of cognitive processing.

Our analysis also identified age and Karnofsky score as major predictors of cognitive dysfunction within the transdermal fentanyl cohort, reflecting previous EPOS findings when patients on any opioid were analysed in combination [28]. However, unlike Kurita and colleagues’ combined analysis [28], there was no major effect of daily opioid dose, lung cancer, time since diagnosis or breakthrough pain, on cognitive dysfunction within the subset of transdermal fentanyl patients included in the present study.

Breakthrough opioid use was more important than serum fentanyl concentrations for predicting adverse symptoms other than cognitive dysfunction. Transdermal fentanyl is typically associated with reduced incidence of nausea, sedation and constipation compared to oral opioids [38]. Therefore, the serum fentanyl concentration-adverse effect relationship may be less pronounced for transdermal fentanyl than for other opioids, with these adverse symptoms influenced more by acute effects of breakthrough opioid use, cancer and/or chemotherapy.

Innate immune activation can also be associated with a general sickness phenotype consisting of symptoms of nausea, tiredness and/or depression [13,14]. However cognitive dysfunction and sickness phenotype were unrelated in this study, with different predictors. No genetic predictors were identified for general sickness phenotype or opioid adverse event complaint, which share common symptoms of nausea and tiredness. Similarly, no genetic variants predicted nausea, tiredness, constipation or depression when analysing these symptoms separately (results not presented). This suggests innate immune activation may play a lesser role in symptoms of nausea, sedation, tiredness and depression compared to cognitive dysfunction, reflecting the differential clustering of these symptoms in cancer patients and thus likely mechanisms [14].

Patients’ pain and adverse effect phenotypes are the summation of the effects of cancer, chemotherapy and opioid treatment. Similarly, cancer, chemotherapy and opioids can all activate the neuroimmune activation pathway being investigated. The major limitation of this study is the cross-sectional design which could not distinguish between underlying (baseline) phenotype and response; that is, between the cancer, chemotherapy, and opioid effects. As discussed above, large variability in baseline (opioid-free) phenotype makes it difficult to establish a serum fentanyl concentration-response relationship across cancer pain patients: repeated measures designs would better demonstrate the concentration-response relationship and aid in separating underlying phenotype from fentanyl response, but are practically and ethically complex in the cancer pain population.

The MMSE was employed for assessing cognitive dysfunction due to its extensive use in research and clinical practice, its gold standard status for measuring cognitive dysfunction in patients with cancer, and a cut-off (a score of 23 or less that has high specificity for defining cognitive dysfunction) that has been applied and validated in cancer patient populations [19,39–43]. However, it is acknowledged that the MMSE has limited sensitivity to detect mild cognitive impairment [44–46], and future prospective studies would be improved by including multiple (validated) assessment tools that help better define mild cognitive impairment and assess specific cognitive domains.

### Future directions

For cognitive dysfunction, the association with age and Karnofsky status, but not serum fentanyl concentrations or breakthrough opioid use, suggest underlying physiology and the impact of the cancer or chemotherapy may be the major contributors to this phenotype. It is probable that the protective effect of the *MYD88* rs6853 variant is due to reduced innate immune activation in response to DAMPS associated with cancer and/or chemotherapy tissue damage, rather than to fentanyl. Therefore, the same association should be seen for other opioids in EPOS; this is currently under investigation in addition to MMSE sub-categories that may point to specific brain regions and processes especially impacted by *MYD88* genetic variability.

The frequency of the rs6853 wild-type genotype (75%) relative to cognitive dysfunction (18%) prohibits a highly sensitive and specific prediction of risk for cognitive dysfunction based on this single SNP. Therefore additional protective or high risk *MYD88* SNPs may remain to be identified. The characterisation of specific MyD88 pathways driving cancer- and chemotherapy-induced cognitive dysfunction will be important, and may point to additional genetic polymorphisms or biomarkers of these pathways useful for identifying patients at high risk of cognitive dysfunction. Further this may prompt the investigation of interventions targeting the MYD88 pathway to ameliorate cognitive dysfunction in cancer patients, similar to those being trialled for post-operative cognitive dysfunction [35].

Whilst candidate SNPs were identified for each response examined in this study, our randomised control analyses demonstrated a high probability that these were chance associations, with the exception of rs6853 for cognitive dysfunction. These candidate SNPs have been reported in supplementary material as they may be true positive associations and subsequently be confirmed in replicate studies.

## Conclusions

Serum fentanyl concentrations were not a major determinant of between-patient variability in effective cancer pain control with transdermal fentanyl, further highlighting the importance and challenge of matching opioid and dose to individual patients from populations of highly heterogeneous pain phenotypes. For adverse symptoms other than cognitive dysfunction, breakthrough opioid use was more important than serum fentanyl concentrations, and they were not predicted by genetic polymorphisms relating to neuroimmune activation pathways. However, the *MYD88* rs6853 variant was associated with significantly reduced risk of cognitive dysfunction, identifying MyD88 signalling pathways as a potential focus for predicting and reducing the burden of cognitive dysfunction in cancer patients.

## Supporting Information

S1 Protocol Statistical analysis pipeline. (DOC)

S1 Table SNP allele and genotype frequencies in EPOS cancer pain patients receiving transdermal fentanyl. (DOCX)

S2 Table Variables associated with sickness response (nausea, tiredness and/or depression complaint) in cancer pain patients receiving transdermal fentanyl. (DOCX)

S3 Table Variables associated with opioid adverse event complaint (as defined by [27]) in cancer pain patients receiving transdermal fentanyl. (DOCX)

S1 Text Supplementary results relating to SNP linkage disequilibrium and haplotypes. (DOC)
