# Supplementary material for: Innate Immune Signalling Genetics of Pain, Cognitive Dysfunction and Sickness Symptoms in Cancer Pain Patients Treated with Transdermal Fentanyl
Source: PLoS One. 2015 Sep 2;10(9):e0137179. doi: 10.1371/journal.pone.0137179 (PMC4557995; doi:10.1371/journal.pone.0137179)
Supplement: S1 Protocol — (DOC) [file pone.0137179.s001.doc]

**S1 Protocol**

This file contains Supplementary Methods (including references) outlining the details of the statistical analysis pipeline for the manuscript entitled “Innate immune signalling genetics of pain, cognitive dysfunction and sickness symptoms in cancer pain patients treated with transdermal fentanyl”.

**Ancestral and non-genetic variables**

Associations between each measure of fentanyl response and ancestral subgroup were checked by chi-squared analysis.

Major non-genetic variables (listed in manuscript Table 1) to be controlled for in subsequent genotype analyses were then identified by Lasso regression (generalised binomial) using cross-validation to identify the appropriate lambda penalty (largest lambda within 1 standard error of the minimum cross-validated error (CVE)) (glmnet and cv.glmnet functions in glmnet package ).

First order interactions between serum fentanyl concentrations and all other variables were included with the aim of specifically identifying the impact of variables on the fentanyl PK-PD relationship.

Co-medications that showed positive associations with an outcome for which they were indicated (e.g. breakthrough opioids for pain relief, or antiemetics for nausea and vomiting) were excluded from analysis. However, where they showed negative associations, they were retained in order to account for their mitigating effects.

Non-genetic variables selected from Lasso regression were then included in all subsequent analyses of genetic polymorphisms.

**Candidate polymorphism analyses**

Only SNPs with minor allele frequencies greater than 5% were included in analyses. LD between SNPs within *IL1B*, *IL10*, *TLR4*, *CASP1* and *ARRB2* was determined using the LD function of the “genetics” package . Where feasible, tightly linked SNPs were combined into haplotypes before further analysis. *ARRB2* haplotypes were inferred using PHASE .

Based on the expectation of modest effect sizes for multiple SNPs, rather than large effects of single SNPs, a step-down regression model selection procedure based on Akaike Information Criterion (AIC) (stepAIC function in MASS package ) was used to identify genetic factors associated with different responses. Fixing non-genetic predictors as the base model, leave-one-out cross-validation was used to identify the optimal k penalty (k ≥ 2) to minimise CVE (cv.glm function in boot package ). If a genetic factor was included at the optimal k (initially using a co-dominance model), different dominance models (wildtype dominant and variant dominant) were tested for model improvement (reduced CVE). If a wildtype or variant dominant model was chosen for a genetic factor, the k optimisation procedure was repeated, and so on until a stable best model was achieved.

Given the number of genetic factors investigated, the likelihood of chance associations within the data for each outcome measure was investigated by comparing the CVE of the final model to the CVE of models generated using 100 permutations of the response (paired to any non-genetic variables) randomised against paired genetic data.

Possible epistatic interactions (excluding *ABCB1* SNPs) were investigated by generalised multifactor dimensionality reduction analysis (Generalised Multifactor Dimensionality Reduction v0.9 ). Phenotype scores were generated within the software using the response measure and any non-genetic variables identified from Lasso regression. Two-, 3-, and 4-way gene interactions were investigated using 5-fold cross-validation for 10 different random seeds. Candidate models within each level were selected based on the following criteria: the highest cross-validation consistency (>20%); median testing balanced accuracy greater than the median + 1 standard deviation testing balanced accuracy of unselected models; and median testing balanced accuracy greater than the median + 1 standard deviation of the highest cross-validation consistency models generated from 10 randomised response data sets.

Receiver operating characteristic (ROC) curves of non-genetic and final models were generated using the “ROC” function of the “epicalc” package , and the effects of each predictor (averaging over other terms in the model) visualised using the “allEffects” function of the “effects” package . Likelihood ratio chi-squared test P-values were obtained using the “Anova” function of the “car” package . Estimates (and 95% confidence intervals) of relative risk were obtained using the “vcovHC” function of the “sandwich” package .

**S1 Protocol References**
