# Supplementary material for: Innate Immune Signalling Genetics of Pain, Cognitive Dysfunction and Sickness Symptoms in Cancer Pain Patients Treated with Transdermal Fentanyl
Source: PLoS One. 2015 Sep 2;10(9):e0137179. doi: 10.1371/journal.pone.0137179 (PMC4557995; doi:10.1371/journal.pone.0137179)
Supplement: S1 Table — (DOCX) [file pone.0137179.s002.docx]

**S1 Table. SNP allele and genotype frequencies in EPOS cancer pain patients receiving transdermal fentanyl.**

| **Gene** | **SNP** | **(a.k.a)** | **Location** | **Nucleotide change** | **Amino acid change** | **Variant allele frequency** | **Genotype n (frequency)** | | |
| --- | --- | --- | --- | --- | --- | --- | --- | --- | --- |
|  |  |  |  |  |  |  | **Wt/Wt** | **Wt/V** | **V/V** |
| *IL6* | rs10499563 | (-6331T>C) | 5' | T>C |  | 0.24 | 264 (0.57) | 184 (0.39) | 19 (0.04) |
| *IL1B* | rs1143627 | (-31T>C) | 5' | T>C |  | 0.32 | 212 (0.45) | 207 (0.44) | 48 (0.1) |
| *IL1B* | rs1143634 | (3954C>T) | exon 5 | C>T | synonymous | 0.25 | 269 (0.58) | 165 (0.35) | 33 (0.07) |
| *IL1B* | rs16944 | (-511C>T) | 5' | C>T |  | 0.32 | 212 (0.45) | 207 (0.44) | 48 (0.1) |
| *LY96* | rs11466004 |  | exon 5 | C>T | Ser157Pro | 0.02 | 446 (0.96) | 21 (0.04) | 0 (0) |
| *OPRM1* | rs1799971 | (118A>G) | exon 1 | A>G | Asn40Asp* | 0.1 | 378 (0.81) | 83 (0.18) | 6 (0.01) |
| *TGFB1* | rs11466314 | (-1287G>A) | 5' | G>A |  | 0 | 468 (1) | 0 (0) | 0 (0) |
| *TGFB1* | rs1800469 | (-509C>T) | 5' | C>T |  | 0.33 | 204 (0.44) | 219 (0.47) | 44 (0.09) |
| *TNFa* | rs1800629 | (-308 G>A) | 5' | G>A |  | 0.15 | 338 (0.72) | 116 (0.25) | 13 (0.03) |
| *IL10* | rs1800871 | (-819C>T) | 5' | C>T |  | 0.23 | 274 (0.59) | 172 (0.37) | 21 (0.04) |
| *IL10* | rs1800896 | (-1082G>A) | 5' | G>A |  | 0.53 | 110 (0.24) | 220 (0.47) | 137 (0.29) |
| *IL2* | rs2069762 | (-330T>G) | 5' | T>G |  | 0.33 | 209 (0.45) | 204 (0.44) | 53 (0.11) |
| *CRP* | rs2794521 | (-717T>C) | 5' | T>C |  | 0.3 | 229 (0.49) | 194 (0.42) | 43 (0.09) |
| *TLR2* | rs3804100 | (1350T>C) | exon 3 | T>C | synonymous | 0.06 | 411 (0.88) | 56 (0.12) | 0 (0) |
| *TLR4* | rs4986790 | (896A>G) | exon 3 | A>G | Asp299Gly | 0.04 | 425 (0.91) | 42 (0.09) | 0 (0) |
| *TLR4* | rs4986791 | (1196C>T) | exon 3 | C>T | Thr399Ile | 0.05 | 423 (0.91) | 44 (0.09) | 0 (0) |
| *CASP1* | rs554344 | (10643G>C) | 3' UTR | G>C |  | 0.17 | 322 (0.69) | 128 (0.27) | 17 (0.04) |
| *CASP1* | rs580253 | (5352G>A) | exon 5/6 | G>A | synonymous | 0.17 | 323 (0.69) | 126 (0.27) | 17 (0.04) |
| *BDNF* | rs6265 | (196G>A) | exon 6 | G>A | Val66Met | 0.21 | 290 (0.62) | 156 (0.33) | 20 (0.04) |
| *MYD88* | rs6853 |  | 3' UTR | A>G |  | 0.13 | 348 (0.75) | 111 (0.24) | 7 (0.02) |
| *IL6R* | rs8192284 |  | exon 9 | A>C | Asp358Ala | 0.37 | 195 (0.42) | 197 (0.42) | 75 (0.16) |
| *COMT* | rs4680 | (472G>A) | exon 4 | G>A | Val158Met | 0.5 | 109 (0.24) | 243 (0.53) | 110 (0.24) |
| *ARRB2* | rs3786047 |  | intron | A>G |  | 0.71 | 44 (0.09) | 186 (0.4) | 235 (0.51) |
| *ARRB2* | rs1045280 | (8622T>C) | exon 11 | C>T | Synonymous | 0.71 | 42 (0.09) | 188 (0.4) | 235 (0.51) |
| *ARRB2* | rs2271167 |  | intron | A>G |  | 0.71 | 43 (0.09) | 185 (0.4) | 233 (0.51) |
| *ARRB2* | rs2036657 |  | 3’ UTR | G>A |  | 0.7 | 42 (0.09) | 189 (0.42) | 223 (0.49) |
| *STAT6* | rs3024971 |  | intron | T>G |  | 0.1 | 373 (0.8) | 88 (0.19) | 4 (0.01) |
| *STAT6* | rs167769 |  | intron | C>T |  | 0.36 | 188 (0.41) | 220 (0.47) | 56 (0.12) |
| *ABCB1* | rs1045642 | (3435C>T) | exon 26 | G>A | Synonymous | 0.51 | 110 (0.24) | 240 (0.51) | 118 (0.25) |
| *ABCB1* | rs2235013 |  | intron |  |  | 0.53 | 96 (0.21) | 238 (0.52) | 124 (0.27) |
| *ABCB1* | rs1128503 | (1236C>T) | exon 12 | G>A | Synonymous | 0.42 | 153 (0.33) | 230 (0.5) | 77 (0.17) |

*Also known as 335A>G Asn102Asp. A.k.a: Also known as.
