# Supplementary material for: Innate Immune Signalling Genetics of Pain, Cognitive Dysfunction and Sickness Symptoms in Cancer Pain Patients Treated with Transdermal Fentanyl
Source: PLoS One. 2015 Sep 2;10(9):e0137179. doi: 10.1371/journal.pone.0137179 (PMC4557995; doi:10.1371/journal.pone.0137179)
Supplement: S2 Table — (DOCX) [file pone.0137179.s003.docx]

**S2 Table. Variables associated with sickness response (nausea, tiredness and/or depression complaint) in cancer pain patients receiving transdermal fentanyl.**

| **Regressor** | **Adjusted Odds Ratio^a^** (95% CI) | | **Nested model Chi-squared**  **P-value** |
| --- | --- | --- | --- |
| **(Intercept)** | 0.67 | (0.45 to 0.99) |  |
| **Any breakthrough opioids in last 24 h** | 1.92 | (1.23 to 3.02) | 0.005 |
| **Italian treatment centre** | 0.26 | (0.15 to 0.44) | 9.2 x 10^-8^ |
| **Sex (male)** | 0.60 | (0.38 to 0.92) | 0.02 |
| ***BDNF* rs6265 variant carrier^b^** | 1.54 | (0.99 to 2.41) | 0.06 |

^a^Odds Ratio controlling for all other regressors. ^b^Homozygous wildtype genotype as reference. Odds ratio greater than 1 indicates an association with increased likelihood of sickness response.
