# Supplementary material for: Innate Immune Signalling Genetics of Pain, Cognitive Dysfunction and Sickness Symptoms in Cancer Pain Patients Treated with Transdermal Fentanyl
Source: PLoS One. 2015 Sep 2;10(9):e0137179. doi: 10.1371/journal.pone.0137179 (PMC4557995; doi:10.1371/journal.pone.0137179)
Supplement: S3 Table — (DOCX) [file pone.0137179.s004.docx]

**S3 Table. Variables associated with opioid adverse event complaint (as defined by [**[**1**](#_ENREF_1)**]) in cancer pain patients receiving transdermal fentanyl.**

| **Regressor** | | **Adjusted Odds Ratio^a^** (95% CI) | | **Nested model Chi-squared**  **P-value** |
| --- | --- | --- | --- | --- |
| **(Intercept)** | | 1.09 | (0.68 to 1.76) |  |
| **Any breakthrough opioids in last 24 h** | | 2.80 | (1.50 to 5.20) | 5.5 x 10^-4^ |
| **EORTC Depression** | | 5.35 | (2.57 to 11.14) | 1.8 x 10^-7^ |
| ***CASP1* rs554344 homozygous variant^b^** | | 5.50 | (0.68 to 44.60) | 0.05 |
| ***ARRB2* “variant” diplotype^b^** | | 1.52 | (0.93 to 2.50) | 0.10 |
| ***TGFB1* rs1800469^c^** | |  |  | 0.11 |
|  | Wildtype/variant | 1.69 | (1.00 to 2.85) |  |
|  | Variant/variant | 1.74 | (0.71 to 4.23) |  |

^a^Odds Ratio controlling for all other regressors. ^b^Combined homozygous wildtype and heterozygous genotypes/diplotypes, and ^c^homozygous wildtype genotype, as reference. Odds ratio greater than 1 indicates an association with increased likelihood of opioid adverse event complaint.

**1.** Klepstad P, Borchgrevink PC, Dale O, Zahlsen K, Aamo T, Fayers P, et al. Routine drug monitoring of serum concentrations of morphine, morphine-3-glucuronide and morphine-6-glucuronide do not predict clinical observations in cancer patients. Palliat Med. 2003; 17:679-87.
