# Supplementary material for: Innate Immune Signalling Genetics of Pain, Cognitive Dysfunction and Sickness Symptoms in Cancer Pain Patients Treated with Transdermal Fentanyl
Source: PLoS One. 2015 Sep 2;10(9):e0137179. doi: 10.1371/journal.pone.0137179 (PMC4557995; doi:10.1371/journal.pone.0137179)
Supplement: S1 Text — (DOC) [file pone.0137179.s005.doc]

**S1 Text**

This file contains Supplementary Results relating to linkage disequilibrium and haplotypes of the SNPs investigated for the manuscript entitled “Innate immune signalling genetics of pain, cognitive dysfunction and sickness symptoms in cancer pain patients treated with transdermal fentanyl”.

**Linkage disequilibrium and haplotypes**

*IL1B* rs16944 was in complete LD with rs1143627, so a single genotype was used to represent these polymorphisms in further analyses. *CASP1* rs554344 and rs580253 were in near complete LD (one patient rs554344 G/G and rs580253 G/G, and one patient rs554344 C/C and rs580253 G/A), therefore only rs554344 was included in further analyses.

*TLR4* rs4986790 and rs4986791 were in strong LD (D’ = 0.97; r^2^ = 0.90), therefore these SNPs were analysed as a two-level factor: dual homozygous wildtype (n = 422) versus other genotype combinations (n = 41 dual heterozygous, n = 3 homozygous wildtype-heterozygous, n = 1 heterozygous-homozygous wildtype, respectively).

*ARRB2* rs3786047, rs1045280, rs2271167 and rs2036657 were all in strong LD (D’ > 0.97; r2 > 0.92) and could be categorised into 3 diplotypes: “Wildtype” (all wildtype genotype, n = 43), “heterozygous” (all heterozygous genotype, n = 182; or at least 2 SNPs heterozygous and remaining wildtype or variant, n = 5), “variant” (all variant genotype, n = 226; or heterozygous for 1 SNP and remaining variant, n = 11). One patient had no genotype results for any *ARRB2* SNP.
